# Case Report: Clinical application of hyperbaric oxygen therapy after bladder fistula repair surgery: a report of 7 cases and literature review

**DOI:** 10.3389/fsurg.2025.1680900

**Published:** 2025-12-10

**Authors:** Anjian Chen, Han Zhu, Pinyao Liang, Shanshan Guo, Tiancai Liang, Faliang Zhao, Zhihui Xie, Guobiao Liang

**Affiliations:** 1 Department of Urology, Affiliated Hospital of Zunyi Medical University, Zunyi, China; 2 Second Department of Urology, The First Affiliated Hospital of Kunming Medical University, Kunming, China; 3 Department of Hyperbaric Oxygen, Affiliated Hospital of Zunyi Medical University, Zunyi, China; 4 Department of Kidney Transplantation, Affiliated Hospital of Zunyi Medical University, Zunyi, China

**Keywords:** vesicovaginal fistula, colovesical fistula, hyperbaric oxygen, bladder fistula, postoperative adjuvant therapy

## Abstract

Hyperbaric oxygen treatment (HBOT) is a therapeutic modality that delivers 100% oxygen under supra-atmospheric pressure, which has been widely used in the clinical management of various diseases. The incidence of genitourinary fistulas is relatively low in developed countries, mainly presenting as complications of gynecological surgeries, while it remains high in developing nations due to factors such as prolonged labor and inadequate medical care. Among these, bladder fistula is the most common type of genitourinary fistula. This article presents the outcomes of 7 patients who received HBOT after bladder fistula repair surgery, including 6 cases of vesicovaginal fistula (VVF) and 1 case of colovesical fistula (CVF). HBOT was initiated on the first postoperative day, administered once daily for a total of 10 consecutive days, and no relevant adverse reactions occurred in any patient during the HBOT course. All patients achieved uneventful postoperative recovery and were discharged from the hospital, with no recurrence of symptoms observed during the 3–12 months of follow-up. As a novel adjunctive therapy for patients after bladder fistula surgery, HBOT exhibits satisfactory preliminary efficacy and favorable safety profile, yet further research with expanded sample size and in-depth investigation is warranted.

## 1 Introduction

### 1.1 Hyperbaric oxygen therapy

Hyperbaric oxygen therapy (HBOT) involves the administration of 100% oxygen at pressures greater than one atmosphere absolute (ATA), typically 1.5–3 times higher than normal. This treatment is delivered in a hyperbaric chamber, where patients breathe pure oxygen at high pressure, increasing the amount of dissolved oxygen in the blood and improving tissue oxygenation. Compared to simple oxygen supplementation, HBOT allows for more oxygen to enter the plasma, thereby achieving therapeutic goals. Its clinical application originated in 1955 when Churchill Davis utilized it to enhance the effects of radiation therapy in cancer patients, followed by its introduction in 1956 by cardiovascular surgeon Ite Boerema for the treatment of surgical patients (1, 2). Over the past 50 years, hyperbaric oxygen has been increasingly used as an adjunctive therapy for various diseases. Its benefits include promoting the healing of chronic wounds, aiding in the treatment of malignant tumors alongside radiotherapy and chemotherapy, facilitating the repair of burn tissue, enhancing periodontal health, and managing conditions such as carbon monoxide poisoning and neurocognitive dysfunction. Moreover, multiple studies have demonstrated the therapeutic effects of hyperbaric oxygen in improving long-term symptoms following COVID-19 infection (3–12).

### 1.2 Definition and classification of bladder fistula

Bladder fistula, also known as vesical fistula, refers to an abnormal channel or opening formed between the bladder and other organs or tissues. These abnormal channels may connect with nearby organs such as the rectum, vagina, uterus, etc., or may communicate with the skin surface. Bladder fistulas are typically caused by trauma, surgical complications, infections, tumors, etc., resulting in rupture or ulcer formation between the bladder wall and adjacent tissues. Common types of bladder fistulas include vesicovaginal fistula (VVF), Enterovesical fistula (EVF), and vesicouterine fistula (VUF) (13).

#### 1.2.1 Vesicovaginal fistula

VVF represents the most prevalent urogenital fistula, with an estimated minimum of 3 million women in impoverished nations suffering from unrepaired VVF, and Africa alone reporting 30,000–130,000 new cases (14). Research indicates that risk factors for VVF formation due to childbirth include primiparity, smaller stature, early marriage, and gynecological surgeries, notably hysterectomy, in developed nations (15, 16). A multicenter study has determined that the incidence of VVF post-benign gynecological surgery stands at approximately 1/1,000, with risk factors encompassing surgical complexity, fibroid size, obesity, substantial blood loss, and smoking (17, 18). Treatment modalities for VVF encompass both conservative approaches (typically for uncomplicated fistulas with a diameter <1.0 cm) and surgical interventions. Surgical methods primarily involve vaginal and abdominal approaches, each tailored to specific patient characteristics (19). Theofanides et al. (20) analyzed data from 200 patients who underwent bladder-vaginal fistula surgery and found that most opted for vaginal repair (65%). Compared to abdominal repair, vaginal repair exhibited a higher success rate, fewer complications, shorter hospital stays, and lower risks of sepsis, blood transfusion, and readmission (21, 22).

#### 1.2.2 Enterovesical fistula

EVF has an incidence of about 1/3,000, with 65%–79% attributed to diverticulitis, most evolving into CVF. Treatment options for CVF include conservative and surgical approaches. While non-surgical treatment may be suitable for specific patients, surgery is often necessary due to the low likelihood of spontaneous closure and the high risk of sepsis (23, 24). However, related studies have also shown that conservative treatment can be considered in patients with benign diseases (25). RVFs, primarily caused by iatrogenic complications, typically necessitate surgical intervention (26).

#### 1.2.3 Vesicouterine fistula

The global rise in cesarean section (CS) rates has led to an increase in VUF, a rare condition accounting for <5% of urogenital fistulas. Conservative treatment's low spontaneous healing rate makes surgery the preferred option, especially for late postoperative complications (27, 28). Surgical techniques vary but predominantly involve fistula excision, partial bladder resection, and uterine reconstruction, with procedures performed through open, laparoscopic, or robotic approaches.

In terms of surgical success rate, taking the most common VVF as an example, Neu et al. conducted a retrospective analysis of 814 patients with VVF, of whom 76% were caused by iatrogenic injuries and 117 cases (14%) required secondary repair. Another study combining VVF and VUF showed that the surgical failure rate of both was approximately 16%, and the main causes of surgical failure included recurrent fistulas, large fistula size, and multiple fistulas (29, 30). To summarize, given the uncertainties and relatively low success rate of conservative treatment, surgery remains the first-choice option for managing vesical fistula in most patients. While conservative treatments have been explored, their effectiveness lacks clear research evidence. The author compiled research reports on conservative treatment methods (Table 1). Against this backdrop, adjuvant therapies aimed at lowering recurrence rates and enhancing surgical outcomes have attracted increasing attention; however, data on the application of HBOT in this context remain surprisingly limited. By delivering oxygen in an environment above atmospheric pressure, HBO therapy effectively promotes tissue oxygenation, angiogenesis, and wound healing, and has demonstrated promising applications in multiple surgical fields such as complex wound repair and anastomotic healing. Nevertheless, its value in preventing fistula recurrence and improving surgical success rates specifically after bladder fistula surgery has not been systematically evaluated.

**Table 1:** Adjuvant treatment of vesical fistula.

Disease	Treatment	Result	Year	References
VVF	Fibrin glue	1/1	1999	Morita (31)
	Platelet-rich plasma	16/16	2019	Dominika (32)
	Laser welding	1/1, 7/8	2001, 2011	Dogra (33, 34)
	Cyanoacrylic glue	11/13	2005	Gioavnni (35)
EVF	Intersegmental antibiotics	3/6	2005	Solkar (36)
	Octreotide	1/1	2009	Shinjo (37)
VUF	Hormonal manipulation	8/9	1999	Jóźwik (27)
	Bladder drainage, antibiotics	1/1	2004	Joseph (38)
	Bladder irrigation, antibiotics, indwelling urinary catheterization	1/1	2020	He (39)

To address this research gap, the present study retrospectively analyzed and summarized clinical data from patients with VVF and CVF who were admitted to our institution and completed follow-up between December 2017 and November 2023. The core objective of this study is to improve the local oxygen supply at the fistula site and inhibit the inflammatory response through hyperbaric oxygen (HBO) intervention, thereby creating favorable conditions for wound healing. It aims to evaluate the efficacy of HBO therapy as an effective adjuvant approach for promoting fistula healing and preventing recurrence after bladder fistula surgery, ultimately providing evidence for optimizing the treatment regimen for patients with bladder fistula.

## 2 Case report

### 2.1 Study subjects and baseline clinical data

In this study, we collected and followed up with seven patients diagnosed with bladder fistula from December 2017 to November 2023, including six cases of VVF and one case of CVF. The age of the patients ranged from 21 to 72 years, with disease durations ranging from 5 to 120 months. Among the VVF cases, four (66.7%) were attributed to obstructed labor and prolonged childbirth. In two cases (33.3%) involving elderly female patients, the etiology of VVF remained undetermined. The single case of CVF occurred in a 47-year-old patient following sigmoid colostomy, presenting with urine-like fluid discharge.

**Figure 1:**
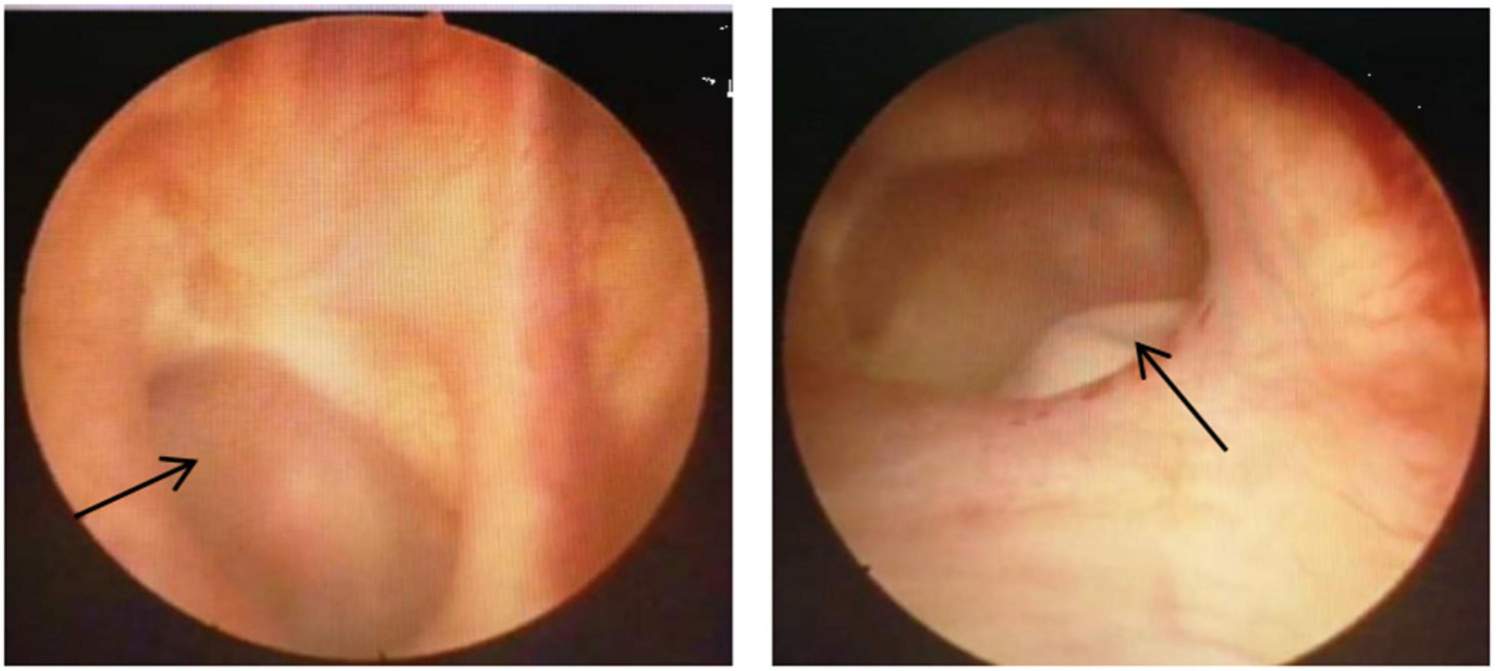
The location of the fistula under cystoscope.

All six patients with vesicovaginal fistula underwent cystoscopy to assess the condition of the fistula. Among the VVF cases, four had single fistulas: two located at the bladder neck (measuring 1.0 cm × 0.8 cm and 0.5 cm × 0.7 cm, respectively), one on the posterior bladder wall (0.5 cm × 0.5 cm), and another beneath the opening of the right ureter (2.0 cm × 3.0 cm). Two patients exhibited complex fistulas, with one positioned above and below the left ureteral ridge (measuring 1.0 cm × 1.0 cm and 0.5 cm × 0.5 cm, respectively), and the other located posterior to both ureter openings (0.4 cm × 0.3 cm and 0.2 cm × 0.3 cm, respectively), one case of VVF located in the posterior wall of the bladder is shown in Figure 1. The single case of CVF manifested a defect in the bladder trigone area detected during surgery, measuring approximately 3.0 cm × 3.5 cm. All patients underwent their initial fistula closure surgery upon admission. The clinical baseline data of the patients are detailed in Table 2.

**Table 2:** Clinical data table of patients with vesical fistula.

Case no.	Age (years)	Type	Possible etiology	Disease duration (months)	Fistula location (size)	Follow-up time (months)
1	46	VVF	Prolonged labor	48	Below the right ureteral orifice (2.0 cm × 3.0 cm)	12
2	21	VVF	Prolonged labor	36	Bladder neck fistula (1.0 cm × 0.8 cm)	6
3	57	VVF	Prolonged labor	120	Bladder neck fistula (0.5 cm × 0.7 cm)	6
4	50	VVF	Prolonged labor	96	Posterior bladder wall fistula (0.5 cm × 0.5 cm)	3
5	72	VVF	Etiology unknown	60	Fistulas above and below the left ureteral crest (1.0 cm × 1.0 cm and 0.5 cm × 0.5 cm)	12
6	61	VVF	Etiology unknown	5	Posterior to bilateral ureteral orifices (0.4 cm × 0.3 cm and 0.2 cm × 0.3 cm)	10
7	47	CVF	Post sigmoid colostomy	9	Bladder trigone fistula (3.0 cm × 3.5 cm)	12

VVF, vesicovaginal fistula; CVF, colovesical fistula.

### 2.2 Preoperative management and surgical procedures

Following the diagnosis of bladder fistula, urinary catheterization was initiated in 7 patients to alleviate urinary leakage symptoms, alongside administration of sensitivity-guided antibiotics based on blood and urine culture results to control infection. For 3 patients with VVF, transvaginal repair surgery was performed. Bilateral ureters were stented with F5 double-J catheters prior to cystoscopic exploration of the bladder to confirm the fistula's location. Subsequent meticulous dissection and excision of bladder and vaginal wall tissues followed, with closure using sutures and iodine irrigation of the vaginal cavity. In 3 other cases of VVF, combined abdominal and transvaginal repair was conducted. A midline abdominal incision facilitated bladder exposure, cystotomy, and ureteral stenting with F5 double-J catheters. Following bladder wall and vaginal wall dissection, layered closure was performed, and a bladder fistulotomy using an F20 mushroom catheter was carried out. Closure of the abdominal cavity ensued. One patient with sigmoid colon bladder fistula underwent bladder fistula repair, sigmoid colon fistula repair, and bilateral ureteral reimplantation. After bladder exposure and cystotomy, excision of scar tissue from the trigone area was performed. Ureteral stenting, bladder wall closure, sigmoid colon fistula excision, sigmoid colon resection, and creation of a colovesical fistula were sequentially conducted.

### 2.3 Hyperbaric oxygen therapy protocol

During the study period (December 2017 to November 2023), patients with absolute contraindications to HBOT were excluded. These contraindications included untreated pneumothorax, severe pulmonary infection, active intracranial hemorrhage, and a previous history of oxygen toxicity. All patients who underwent vesical fistula repair received HBOT as a standardized adjuvant therapy. Postoperatively, all patients received hyperbaric oxygen therapy once daily for 10 days at 2.0–2.4 atm, leading to significant symptom improvement and discharge. Postoperatively, all patients received a standardized hyperbaric oxygen therapy (HBOT) course, with specific therapeutic details as follows:

The entire HBOT regimen consisted of one treatment course, totaling 10 days, and adopted an “intermittent treatment mode” to balance efficacy and patient tolerance: patients first received HBOT for 5 consecutive days, then took a 2-day rest to reduce potential physiological load, and finally completed the remaining 5 consecutive days of treatment. All treatments were performed using a GY-type multi-person hyperbaric oxygen chamber. Daily treatment parameters were as follows: (1) Treatment frequency: Once daily, with a fixed treatment cycle to ensure stable therapeutic effects. (2) Pressure control: During the formal oxygen inhalation phase, the pressure inside the chamber was stably maintained at 2.0–2.4 absolute atmospheres, a commonly used effective pressure range for postoperative rehabilitation. (3) Time allocation: Each single treatment session included three key stages: ① Pressurization phase: 20–30 min, during which the chamber pressure was gradually increased to the target range (2.0–2.4 atm) to avoid discomfort caused by sudden pressure changes; ② Oxygen inhalation phase: 60 min, the core therapeutic stage, during which patients inhaled high-concentration oxygen under the maintained target pressure to promote tissue oxygenation and repair; ③ Depressurization phase: 30–40 min, during which the chamber pressure was slowly reduced to atmospheric pressure to prevent complications such as decompression sickness.

During the entire treatment process, none of the patients experienced HBOT-related adverse reactions or intolerance, and no adverse events occurred, indicating good treatment safety.

### 2.4 Postoperative follow-up and outcomes

**Figure 2:**
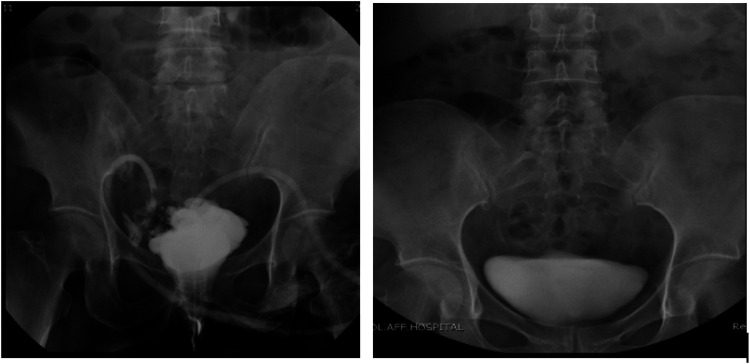
The cystography of 1 case of VVF treated with hyperbaric oxygen.

Ureteral stents and urinary catheters were routinely removed during outpatient follow-up 1 month later. Follow-up assessments demonstrated favorable outcomes, with no recurrence of symptoms in VVF patients and successful resolution of fistula leakage in the sigmoid colon fistula patient. The cystography of 1 case of VVF treated with hyperbaric oxygen is shown in Figure 2. This detailed and comprehensive approach, coupled with hyperbaric oxygen therapy, underscores the efficacy of bladder fistula repair procedures.

## 3 Discussion

Hyperbaric Oxygen Therapy (HBOT) has been widely applied as an adjuvant therapeutic modality in clinical practice for various diseases, particularly hypoxic diseases, ischemic/ischemia-reperfusion injury-related diseases, inflammatory diseases, and trauma with tissue repair disorders. For instance, in the field of ischemia-reperfusion injury, HBOT has demonstrated definite therapeutic effects on ischemia-reperfusion injury of various organs (40–43). In the field of chronic inflammatory diseases, accumulating evidence from recent studies has confirmed that HBOT can not only significantly improve the overall prognosis of patients with inflammatory bowel disease (IBD) but also exhibits prominent advantages in the treatment of Crohn's disease (CD) complicated with perianal fistulas. As an adjuvant treatment, HBOT can significantly optimize the clinical outcomes of such patients, and even achieve fistula healing in some patients with severe and refractory perianal Crohn's disease (44–46). Notably, our own experience and several studies have underscored its significance in managing urological conditions (2).

For instance: Radiation-induced hemorrhagic cystitis (RHC): Radiation therapy, commonly used in treating pelvic malignancies like prostate and bladder cancer, can induce inflammation in nearby arterial intima and increase small blood vessel fragility, leading to bleeding. HBOT has demonstrated efficacy in managing post-radiation hematuria and associated symptoms, albeit its effectiveness may diminish over time (47–49). Interstitial cystitis/painful bladder syndrome (IC/PBS): Characterized by bladder pain and pathological bladder manifestations linked to urinary frequency, IC/PBS presents treatment challenges due to unclear etiology. Multiple studies suggest that HBO may normalize healing processes in damaged bladder tissue and restore balance to defensive factors, thereby ameliorating symptoms (50, 51). Acute kidney injury (AKI): AKI, a heterogeneous condition marked by a sudden decline in glomerular filtration rate, often presents as increased serum creatinine concentration or oliguria (52). Previous animal model studies indicate that HBOT may confer renal protection in ischemia-reperfusion injury by inhibiting inflammatory responses, enhancing autophagy, reducing apoptosis of renal tubular epithelial cells, and mitigating the release of oxygen free radicals and related inflammatory mediators. Additionally, it may decrease the expression of apoptosis proteins PERK and C/EBP homologous protein (CHOP) in endoplasmic reticulum stress, thus exerting renal protective effects (53–56). Male infertility and testicular injury: Animal model research suggests that HBO can mitigate testicular injury under hypoxic conditions and improve male fertility (57–59). Malignant tumors: Several studies indicate that HBOT can potentiate radiotherapy's efficacy against hypoxic cancer cells. Consequently, concurrent radiotherapy during hyperbaric oxygen breathing may reduce mortality and recurrence rates, especially in head and neck malignancies (60). However, further research is warranted to confirm its efficacy in urological tumors. This study has summarized HBOT's role in urological surgery in Table 3.

**Table 3:** Clinical studies of HBO in urology.

Disease/References	Experimental subject	Number	Mode of HBO sessions	Result (PR/CR)	Year
RHC					
Oscarsson (47)	Humans	40	2.4–2.5 atm, 90 min/session, 30–40 times/total	*N* = 29 (73%)	2019
Cardinal (48)	Humans	602	2.0–2.5 atm, 60–95 min/session 30–33 times/total	*N* = 506 (84%)	2018
IC/PBS					
Gallego (50)	Humans	7	2.5 atm, 60 min/session, 20 times/tota	*N* = 7 (100%)	2013
Minami (51)	Rats	4	2.0 atm, 30 min/session, on days 4 and 7	HR	2018
Testicular torsion					
Kolski (57)	Rats	8	2.4 atm, 60 min/session, 2 sessions/day	HR	1998
AKI					
Ostojic (61)	Rats	16	2.0 atm, 60 min/session, 2 sessions/day	HR	2021
Kovacevic (62)	Rats	27	2.0 atm, 60 min/session, 2 sessions/day	HR	2020

HBO, hyperbaric oxygen; RHC, radiation-induced hemorrhagic cystitis; IC/PBS, interstitial cystitis/painful bladder syndrome; AKI, acute kidney injury; HR, histological recovery.

HBOT provides multi-dimensional regulatory support for bladder fistula healing, and its mechanism of action is closely associated with the core molecular regulatory network of tissue repair. In terms of oxygen supply optimization, HBOT significantly increases arterial partial pressure of oxygen via inhalation of pure oxygen in a hyperbaric chamber. It delivers sufficient oxygen to the hypoxic tissues around the bladder fistula, which are caused by surgical trauma, infection, or local ischemia, reverses the hypoxic microenvironment, and lays a foundation for cell proliferation and tissue repair (63). Regarding vascular regeneration regulation, HBOT can upregulate the transcription and expression of pro-angiogenic factors such as vascular endothelial growth factor (VEGF), promote neovascularization of capillaries around the fistula and reconstruction of collateral circulation, and improve the efficiency of local microcirculation and nutrient transport (64, 65). In addition, HBOT can significantly inhibit the release of core pro-inflammatory factors including tumor necrosis factor-α (TNF-α) and interleukin-1β (IL-1β), while blocking the activation of the nuclear factor-κB (NF-κB) inflammatory signaling pathway, thereby reducing local inflammatory responses and creating a stable microenvironment for bladder fistula repair (66–69).

This study has several limitations: ① Small sample size with uneven subtype distribution, including only 7 patients (6 cases of vesicovaginal fistula and 1 case of colovesical fistula), without covering subtypes such as rectovesical fistula and vesicouterine fistula, which limits the extrapolation of results; ② Lack of a control group receiving only surgical treatment, making it impossible to confirm the independent efficacy of hyperbaric oxygen therapy, which requires further verification by subsequent randomized controlled trials; ③ Potential biases exist, including possible selection bias in enrolled patients, and insufficient follow-up time (<6 months) in some patients, which may lead to missed long-term recurrence.

## 4 Conclusion

Hyperbaric oxygen therapy emerges as a promising modality to enhance the success rate of bladder fistula surgery and mitigate postoperative recurrence. Its efficacy as a standalone intervention underscores its potential significance in clinical practice and positions it as a crucial component of postoperative rehabilitation for bladder fistula patients. Further exploration and wider implementation of HBOT in this context hold considerable promise for optimizing patient outcomes and advancing treatment strategies.

## Data Availability

The original contributions presented in the study are included in the article/Supplementary Material, further inquiries can be directed to the corresponding authors.
